# Standard Correction of Vision Worsens EMG Activity of Pericranial Muscles in Chronic TMD Subjects

**DOI:** 10.1155/2020/3932476

**Published:** 2020-04-13

**Authors:** Annalisa Monaco, Eleonora Ortu, Mario Giannoni, Pierdomenico D'Andrea, Ruggero Cattaneo, Alessandra Mummolo, Davide Pietropaoli

**Affiliations:** MeSVA Department, University of L'Aquila, P.le Salvatore Tommasi 11, 67100 L'Aquila, Italy

## Abstract

Recent studies showed an evident correlation between the stomatognathic system and the visual system. These results suggest that subjects who are affected by both temporomandibular (TMD) disorders and refractive disorders present with altered control of pericranial musculature tone and higher open-eye electromyographic (EMG) values. The objective of this work was to evaluate the effects of standard vision correction on EMG in subjects suffering from TMD compared with application of the same vision treatments to non-TMD subjects. 40 subjects were enrolled in this study. The test group included 20 myopic subjects and also included patients with TMD. The control group included 20 healthy myopic subjects. All of the participants underwent a complete ocular examination and a sEMG analysis. The results showed that TMD subjects with vision disorders that are corrected with standard glasses present EMG values that are significantly higher than those presented by non-TMD subjects with vision disorders and standard glasses. Infact, in TMD subjects, eye correction did not have a positive effect on the stomatognathic or pericranial musculature.

## 1. Introduction

A correlation between the stomatognathic system and the visual system has been suggested on the basis of clinical and instrumental observations. For example, it has been observed that myopia occurs more frequently in subjects with second class 1^st^ division, while astigmatism is more characteristic in subjects with a crossbite [1]. However, from an electromyographic (EMG) point of view, opening of the eyes frequently determines alterations in signals that occur at the level of the stomatognathic musculature [2]. It is also interesting to note that for children with functional lateral deviation, there are data that indicate that the correction of vision defects with standard eye techniques can lead to an increase in EMG values of the stomatognathic musculature [3]. In healthy adults that are not affected by temporomandibular disorders (TMD) and sight defects, EMG values of their stomatognathic system and neck muscles do not show significant variations if taken when the individuals have their eyes closed versus open [4]. These observations indicate that visual input, *per se*, does not induce an increase or a significant change in the electric activity of the muscles correlated to the stomatognathic system in healthy individuals. In contrast, under rest conditions and with their eyes closed, adult individuals suffering from masticatory muscle pain show an increase in their EMG activity [5]. While such feedback appears to be of greater statistical importance than clinical importance, there are data that suggest that these values are higher when eyes are open [6]. Therefore, the characteristic of individuals with myogenic pain appear to be related to difficulties in adapting to variations in their visual input rather than absolute values of a single test or condition (e.g., eyes closed or open). In general, we propose that a relationship exists between refractive disorders, vision disorders, the stomatognathic system, and EMG hyperactivity, as described in a recent literature review [7]. It has been reported that the prevalence of vision disorders in the general population is high, and it is increasing in Europe and elsewhere worldwide [8, 9]. There is also a high prevalence of TMD in the general population (affecting approximately 5–20% annually) [10]. Moreover, for those in the general population who suffer from headaches, these individuals have up to a 15-fold higher probability of developing TMD compared with the general population [11].

Refractive disorders are often associated with muscular disorders involving eye movement. Correspondingly, individuals suffering from refractive and oculomotor dysfunctions tend to be more affected by headaches than individuals who are not affected by such disorders [12–14]. Furthermore, individuals with tension headaches present with higher EMG pericranial muscular activity than individuals who do not suffer from tension headaches, similar to individuals who suffer from TMD and exhibit a high incidence of tension headaches [15–18]. The above considerations suggest that subjects who are affected by both TMD and refractive disorders present with altered control of pericranial musculature tone and higher open-eye EMG values. To the best of our knowledge, the influence of standard vision correction on the activity of pericranial and stomatognathic muscles in TMD subjects has not been investigated. Thus, we hypothesize that the state of muscular activation induced by opening of the eyes, especially in TMD subjects, is clinically relevant to investigations of the effects of standard vision correction on EMG. Therefore, the aim of this work was to evaluate the effects of standard vision correction on EMG in subjects suffering from TMD compared with application of the same vision treatments to non-TMD subjects.

## 2. Materials and Methods

### 2.1. Selection of Subjects

This study was carried out in accordance with the fundamental principles of the Declaration of Helsinki and was approved by the Internal Review Board (IRB) of the University of L'Aquila (Number 16137/2016). Written informed consent was obtained from all the participants. A total of 75 myopic patients and patients with corrective glasses for myopia (40 females and 35 males) with a mean age of 27 ± 1.5 years were examined by the same dentist. Next, all of the subjects underwent an eye examination that was conducted by an expert ophthalmologist who was blinded to the TMD subjects and control subjects, as well as the purpose of the visits. In order to reduce operators bias, the same calibrated dentist performed TMD diagnosis (RC) according to DCTMD. According to DC/TMD, the enrolled patients had myofascial pain and TMD pain [19]. Subjects who had a discrepancy between eye standards and the glasses they were wearing, subjects with systemic diseases, as well as epileptic subjects, were excluded from this study. Consequently, a total of 40 subjects were enrolled in this study, and the test group included 20 myopic subjects. The glasses worn by the latter subjects were verified by an ophthalmologist to be correct. The test group also included patients with TMD, based on diagnostic criteria (DC) [19]. The control group included 20 healthy myopic subjects (10 males and 10 females) with a mean age of 25 ± 2 years.

All of the participants in this study underwent a complete ocular examination that consisted of a slit-lamp bio-microscopy, a fundus examination, and an evaluation of intraocular pressure. The best-corrected visual acuity (BCVA) for all of the participants was 10/10. In addition, Snellen and ophthalmologic examinations were normal for all of the participants. All of the subjects completed a central sensitization inventory (CSI) questionnaire and subsequently underwent an EMG examination and a sEMG analysis. SCAN 9 with muscle tone was evaluated with the subjects' eyes closed with and without glasses and with the subjects' eyes open with and without glasses in order to identify specific muscle activity (e.g., masseters, anterior digastrics, sternocleidomastoid, and anterior temporalis) in a resting position.

### 2.2. Eye Standard

Eye standard corresponds to the ability of the eye to see distinctly within a field of vision a figure placed at a given distance. Visual acuity is evaluated by recognizing signs or symbols called Snellen's optotypes or tables. A subject must recognize a set of letters and the subject's vision is determined based on the ratio of the size of the letters correctly read to the size of reference letters (in the European system, the size of the symbols in the 10^th^ row is used as reference letters). Visual acuity is inversely proportional to the height of the alphabetical letters read. For example, 1/10 visual acuity is conventionally considered to correspond to the ability to read a letter size of 75  mm at a distance of 5  m. For 10/10 visual acuity, the size of the letters is 7.5  mm. In general, a young subject can see 5  mm letters at a distance of 5  m [20, 21].

### 2.3. Electromyography Instrumentation

EMG activity was recorded with an eight-channel Myotronics K7 Evaluation System (Seattle, WA, USA) equipped with bipolar electrodes with an interelectrode distance of 20  mm. Before positioning the electrodes, each patient's skin was thoroughly cleaned with alcohol. Electrodes were positioned on the left and right masseter muscles (LMM and RMM, respectively) and on the left and right anterior temporal muscles (LTA and RTA, respectively), as described by Castroflorio et al. [22, 23]. Electrodes were also placed on the left and right anterior digastric muscles (RDA and LDA, respectively) [24] and on the left and right sternocleidomastoid muscles (LSC and RSC, respectively) bilaterally parallel to the muscular fibers and over the lower portion of the muscle to avoid the innervation point, as described by Falla et al. [25].

Electrical signals were amplified, recorded, and digitized with the K7 clinical software package (Myotronics Inc., http://www.myotronics.com/). Root mean square (RMS) values (in µV) were used as indices of signal amplitude. Each EMG epoch lasted 15  s. Muscle tone (SCAN 9) was evaluated with eyes closed with and without glasses and with eyes open with and without glasses. It should be noted that the subjects were instructed about the tests they were to complete and that they needed to open their eyes without forcing their eyelids, wrinkling their forehead, or squeezing their eyes. The EMG test was carried out only after the subjects had repeated the tests and demonstrated that they were able to perform the test correctly [26, 27].

Here is a summary of the protocol used:

1. Subject closes eyes and is without glasses. The third screen in which the values are stable is used to calculate average values. No function artifacts (e.g., swallowing) or movement should occur.
2. Subject is asked to open their eyes (they are still without glasses) when they reach approximately halfway down the screen that was shown in (1). An operator ensures that opening of the eyes has occurred without wrinkling of the forehead or visible displacement of the head.
3. At this point with the subject's eyes open, the following period is considered valid. During the open-eye test, the subject is invited to read silently the first, second, and third lines, with the subsequent lines covered, of a standard Snellen optotype. The subject is positioned 3  m away from the optotype, which corresponds to vision acuity of 1/10, 2/10, and 3/10, respectively.
4. The sequence from 1 to 3 is subsequently repeated with the subject's glasses on. Then, the subject is asked to silently read the fifth, sixth, and seventh lines (with the remaining lines covered), which corresponds to vision acuity of 5/10, 6/10, and 7/10, respectively.

The level of optotype reading and the distance from the subject (less than 5  m) were chosen so that the subjects would not be expected to perform at the maximum capacity of their vision.

### 2.4. DC

Research Diagnostic Criteria for Temporomandibular Disorders (RDC/TMD) were previously proposed by Dworkin and LeResche for research efforts regarding orofacial pain [28]. We applied an updated version of these criteria, DC/TMD [19, 28, 29], to clinical data in the present study.

### 2.5. CSI

To evaluate central sensitization, such as cutaneous allodynia and hyperalgesia in the trigeminal and extratrigeminal areas, a CSI was administered. Central sensitization (CS) is a proposed physiological phenomenon in which neurons of the central nervous system become hyperexcitable, thereby resulting in hypersensitivity to both noxious and nonnoxious stimuli [30].

Central sensitivity syndrome (CSS) describes a group of medically indistinct (or nonspecific) disorders, such as fibromyalgia, chronic fatigue, and irritable bowel, for which CS may be a common etiology. Our patient's CSI total score was 55.4, thereby revealing a severe form of central sensitization [30, 31].

### 2.6. Statistics

Acquired data were tested for normality with the Shapiro–Wilk test as a parametric approach. Single muscle sEMG activity was calculated for the healthy and TMD groups according to experimental settings (e.g., eyes closed (EC), eyes open (EO), eyes closed with glasses (ECWG), and open eyes with glasses (EOWG)). Total sum of sEMG activity was derived in the same manner. A paired *t*-test was used to compare between experimental settings, while an unpaired *t*-test was used to compare differences between groups. Differences in CSI were also tested with an unpaired *t*-test. Statistical significance was set at *p* < 0.05. All of the statistical analyses were performed with public domain R libraries. Plots were generated with the “ggplot” library.

## 3. Results

**Figure 1:**
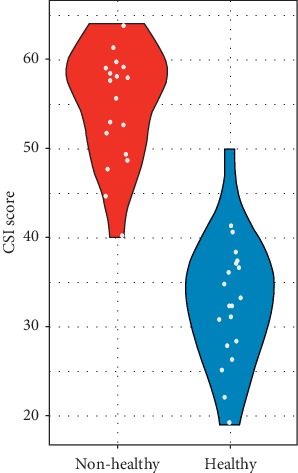
Distribution of CSI scores for the TMD (red) and healthy (blue) groups.

Table 1 and Figure 1 show the CSI values obtained for the TMD and control subjects in our cohort. The average values for the TMD subjects are significantly higher than those of the control subjects (55.40 vs. 32.95, respectively). Values greater than 50, which were received by the TMD subjects, are indicative of severe CS status. Meanwhile, values less than 40 are indicative of a normal or sub clinical status of CSI. There were no significant differences observed between the male and female subjects within each group.

Intergroup comparisons between the EMG values of individual muscles for EC versus EO conditions. No significant differences were observed among the two groups (Table 2).

Table 3 presents the sum of the sEMG values recorded for our cohort. The sum of the EMG values was significantly higher for TMD subjects that had their eyes open versus closed (18.88 vs. 20.55, respectively; *p* ≤ 0.001). In contrast, the difference in EMG values between the control subjects with their eyes open versus closed was not significant (12.84 vs. 12.95, respectively).

When EMG values were compared for closed eyes with and without glasses, there were no significant differences in the TMD and control groups (Table 4).

There was also no significant difference in the sum of EMG values between the subjects with their eyes closed with and without glasses for both the TMD and control groups (Table 5).

Table 6 shows the EMG values obtained for individual muscles between the TMD and normal groups that were evaluated with eyes open with and without glasses. Significantly higher values were obtained for the TMD subjects wearing glasses for all of the muscles examined (e.g., LTA, RTA, LSM, RSM, LDA, and RDA), with the exception of the right and left masseter muscles (LMM and RMM). Meanwhile, in control subjects, none of the muscles exhibited significantly different EMG values with or without glasses.

Furthermore, the sum of EMG values for the individual muscles was significantly greater with eyes open with glasses than without glasses in the TMD group (20.55 vs. 27.61, respectively). Conversely, the sum of the EMG values of the individual muscles was significantly lower in the control subjects group with and without glasses (12.95 vs. 12.48, respectively), and there was no significant difference (Table 7).

**Figure 2:**
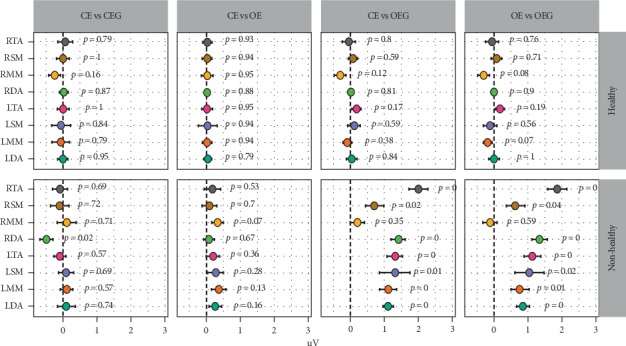
Results of electromyographic values in the two groups.

In Figure 2, a visual representation of the individual muscle data described above for the TMD and control groups are presented. Differences between the two groups are particularly obvious for the EC and EO versus EOWG for the two groups.

Thus, the following observations were made based on the data analyzed:

1. For all of the tested conditions, the TMD subjects exhibited significantly higher EMG values than the control subjects.
2. The TMD subjects showed significant increases in EMG values between closed eyes and open eyes without glasses, and more so between open eyes with and without glasses.
3. The control subjects did not show significant increases in their EMG values between closed eyes and open eyes without glasses, yet the EMG values were lower when the subjects' eyes were open and glasses were worn. The latter data contrast with the corresponding data for the TMD subjects.
4. The CSI values were significantly higher for the TMD subjects compared to the control subjects. Moreover, the former values are indicative of a serious CS condition, while the values for the control subjects indicate a normal or subclinical CS condition.

**Figure 3:**
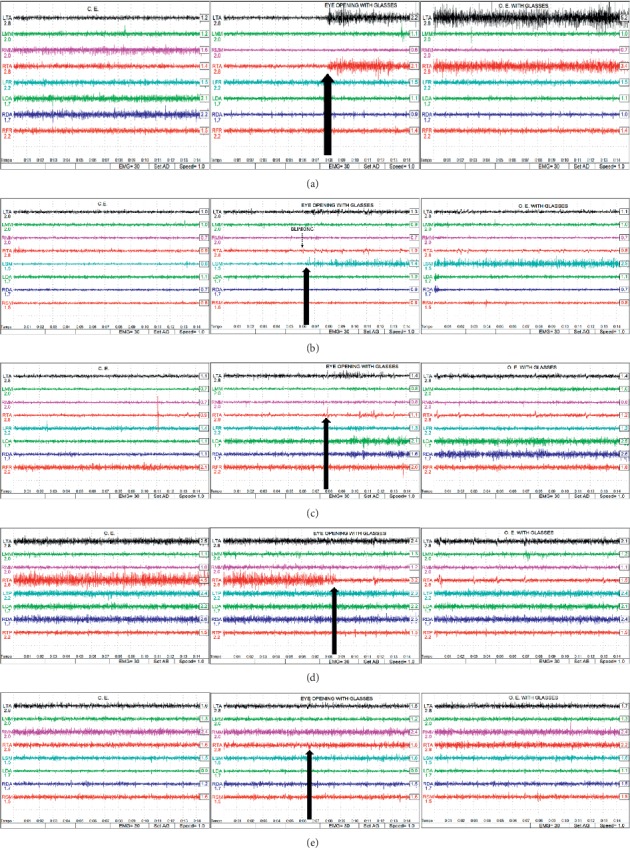
(a–e) Five different cases involving detection of EMG reactions upon opening of the eyes. (a) A trace representing a bilateral increase in an EMG signal from the anterior temporal musculature. (b) A trace representing a monolateral increase in the EMG signal from the left sternocleidomastoid. (c) A trace representing a bilateral increase in the EMG signal from the sovraioidea musculature (anterior abdomen of the digastrics). (d) A trace representing a monolateral decrease in the EMG signal from the RTA. (e) A trace representing the EMG signal from opening of the eyes.

Finally, the electromyographic traces are shown in Figure 3(a)–3(e).

## 4. Discussion

The data obtained in this study indicate that TMD subjects with vision disorders that are corrected with standard glasses present EMG values that are significantly higher than those presented by non-TMD subjects with vision disorders and standard glasses. While the relationship between EMG and TMD remains a topic of debate, our results are consistent with those previously reported, which suggest that resting EMG data of subjects suffering from TMD differ from control subjects [5, 32–34]. However, it should be noted that some authors have indicated that EMG data are insufficient for clinical and research purposes [35]. Discrepancies can arise due to differences in techniques and tools of analysis, study protocols, and in the selection of subjects. Yet, regardless of the reliability of surface EMG values for a diagnosis of TMD, their application in the present study was not to support a diagnosis of TMD, but rather they were used to examine a possible direct correlation between variations in EMG values from opening of the eyes, which, in most cases, is an easily observed phenomenon on EMG traces as shown in the figures.

In the present study, a bilateral increase from the anterior temporal musculature, a monolateral increase from the left sternocleidomastoid, and a bilateral increase from the sovraioidea musculature (anterior abdomen of the digastrics) were more frequently observed in the TMD subjects, while a monolateral decrease from the RTA and from opening of the eyes were observed in the control subjects. These results are consistent with accumulating evidence that indicates that subjects suffering from TMD exhibit dysregulation of the systems that control the response of the autonomous and somatomotor systems to sensory stimuli [36]. Furthermore, it has been hypothesized that this dysregulation in TMD patients represents a form of CS [37–41]. In fact, our CSI data indicate that TMD subjects receive high scores, and this is consistent with a role for central involvement that is not observed in control subjects. In previous studies of central dysregulation in TMD, a notable observation is that the pupil system of TMD subjects responds in a dysregulated manner to teeth clenching in response to administration of ultra-low-frequency transcutaneous electrical nervous stimulation (ULF-TENS) with sensory amplitude, which represents a central action mechanism [27, 37, 42–44]. In the present study, opening of the eyes was found to have a significant effect on EMG values only when TMD subjects opened their eyes while wearing glasses that provided standard eye correction. In contrast, EMG values for healthy subjects and from tests conducted with the eyes open without glasses did not significantly vary. Considering that in the protocol used, there were no differences observed between the two groups in terms of epidemiology or type of vision disorder, and that all subjects had a vision correction that was confirmed to meet ophthalmological standards, the only variable that differentiates the two groups is a TMD condition. The TMD subjects, on average, exhibited an increase in EMG values from the pericranial muscles upon opening of the eyes, and when the same subjects were wearing corrective glasses, the increase was even greater.

The aforementioned effect cannot be considered peripheral because possible connections of the visual/oculomotor system are not directly related to the position of the jaw and to the activity of the investigated stomatognathic muscles. Such relationships may be indirect and require a circuit that leads from sight and ocular motion centers to the nuclear trigeminal complex, and further to trigeminal/hypoglossal/facial motor nuclei (e.g., a central circuit). In animal studies, projections from the superior colliculus were observed to spread to a large part of the trigeminal sensory complex [45, 46]. The superior colliculus plays an important role in the orientation of the head and the eyes towards a salient visual stimulus [47, 48], it has a critical role in managing the visual structures of the neck and face in response to an unexpected object in the visual field, and it plays an important role in generating saccades and in the mechanisms of object tracking [49, 50]. Correct functioning of collicular transmission is, in part, related to structures that govern the state of attention and arousal [50]. At the same time, the superior colliculus receives somatosensory afferents from neurons in the main and spinal trigeminal nucleus that are connected to various somato-sensory orofacial structures [51].

The relationships that have been identified between the superior colliculus and the trigeminal nuclear complex suggest that these two nervous structures collaborate in organizing defensive and behavioral responses and appropriate control of motor responses to intercept and evaluate objects that appear, even suddenly, in the visual field. To optimize one's ability to discriminate sensory stimulation, to interpret its meaning and value, and to respond with a valid motor behavior with an adequate state of arousal is indispensable. This arousal state is partly modulated by central structures, including catecholaminergic nuclei which are present in the trunk of the brain and in reticular formations and the locus coeruleus [52]. Tonic and phasic discharge modes of the locus coeruleus, in particular, have been associated with the behavioral states of arousal and hyperarousal. In the latter case, an altered coupling between a sensory somato/sensory stimulus and an adequate motor and behavioral response is possible [53, 54]. Activity of the pupil muscles is related to that of the locus coeruleus [55], and both are related to exploration activity and attention to an environment [56]. Furthermore, the activity of noradrenergic arousal systems can influence the phasic tonic activity of muscles that are innervated by trigeminal and hypoglossal nuclei [56–58].

Voluntary teeth clenching and rubber mastication in humans can modify pupil dynamics, probably indirectly, via stimulation of the locus coeruleus and the ascending systems of arousal [59]. Alterations in occlusal status or chronic pain disorders of the trigeminal region are associated with dysregulation of arousal-related systems [42, 60]. By 1949, Moruzzi and colleagues demonstrated the importance of trigeminal afferents in maintaining an arousal status, and these afferents were later shown to be essential for proper functioning of the arousal system. Projections from the main spinal and mesencephalic nucleus of the trigeminal to the various noradrenergic nuclei of the encephalic trunk are widespread, and they are responsible for modulation of the arousal state through an ascending reticular activating system and through structures that control the tonic state of the musculature of the head, neck, and ocular motion [61–64].

TMD, as previously noted, are categorized as CSSs and are sometimes referred to as hyperarousal syndromes which are characterized by dysregulation of the arousal state and difficulty in responding adequately to visceral somatosensory stimuli [65, 66]. This categorization suggests the existence of an altered response mode to peripheral inputs, including those coming from the visual system where the balance between visual and trigeminal structures can be altered. In this case, and when arousal systems are dysregulated, it is possible that standard vision correction induces an exaggerated activation in some TMD subjects due to CS. This activation could be interpreted as overstimulation of the associated trigeminal areas, and their response could result in an unbalanced activation of tone for muscles that are innervated by the trigeminal nuclear system. The observation in the present study that the control subjects had lower EMG values and improved quality of visual sensory information when opening their eyes with standard vision correction is consistent with our original hypothesis. Furthermore, these data can be attributed to the best visual acuity for the subjects when interpreting the “nature” that surrounded them because it favors the selection of salient stimuli, which is typical of a state of arousal suitable to a no-alarm circumstance as occurred during the study session.

## 5. Conclusion

A limitation of the present work is that a speculative hypothesis mainly based on indirect data obtained from the literature is used to explain phenomenon observed at the EMG level. However, research data from various fields are consistent with the hypothesis that chronic TMD belongs to a broad category of chronic pain disorders in which pathogenesis of a central type, rather than a peripheral type, plays a key role. Thus, the present work supports an indirect relationship of a probable central nature between visual and stomatognathic musculature that are not directly related to one another. Another limitation of the present study is that the number of analyzed subjects is not large. We applied strict inclusion criteria to obtain TMD patients and healthy subjects that were only affected by myopia and not other types of vision disturbances. Consequently, it is possible that our results are influenced by this selection and that the results may differ for TMD subjects with other vision pathologies. However, our goal was not to create a new nosography that correlates visual defects with the behavior of the stomatognathic musculature or to demonstrate a differentiated impact based on the visual defect associated with TMD. Rather, the aim of the present study was to test the hypothesis that correction of vision according to ophthalmological standards improves tone of the stomatognathic musculature. In healthy subjects, this hypothesis was confirmed. However, in TMD subjects, eye correction did not have a positive effect on the stomatognathic or pericranial musculature. Thus, it remains to be evaluated whether the EMG activity of TMD subjects can be improved with “nonstandard” visual corrections.

## Figures and Tables

**Table 1:** CSI scores.

Group	CSI TMD value (±SD)	CSI healthy value (±SD)	*p* value
Total	55.4 (6.62)	32.95 (7.22)	≤0.001
Female	56.4 (7.35)	32.8 (7.11)	≤0.001
Male	54.4 (6.02)	33.1 (7.72)	≤0.001
Female vs. male	0.92	0.51	

**Table 2:** EMG values for specific muscles in the TMD and healthy subjects with eyes open (EO) versus eyes closed (EC).

Muscle group		EC			EO				
		*N*	Mean	SD	*N*	Mean	SD	*t* value	*p* value
TMD group	LTA	20	2.65	0.5	20	2.83	0.7	0.936	0.355
	RTA	20	2.78	0.8	20	2.94	0.8	0.632	0.531
	LMM	20	2.02	0.6	20	2.37	0.8	1.565	0.126
	RMM	20	2.21	0.5	20	2.53	0.6	1.832	0.075
	LSM	20	2.36	0.8	20	2.62	0.7	1.094	0.281
	RSM	20	2.87	0.6	20	2.95	0.7	0.388	0.700
	LDA	20	2.01	0.5	20	2.26	0.6	1.431	0.160
	RDA	20	1.98	0.4	20	2.05	0.6	0.434	0.667

Healthy group	LTA	20	1.54	0.5	20	1.55	0.5	0.063	0.950
	RTA	20	1.63	0.4	20	1.64	0.3	0.089	0.929
	LMM	20	1.78	0.4	20	1.79	0.4	0.079	0.937
	RMM	20	1.81	0.6	20	1.82	0.5	0.057	0.955
	LSM	20	1.95	0.8	20	1.97	0.9	0.074	0.941
	RSM	20	1.85	0.3	20	1.86	0.5	0.077	0.939
	LDA	20	1.13	0.3	20	1.16	0.4	0.268	0.790
	RDA	20	1.15	0.2	20	1.16	0.2	0.158	0.875

**Table 3:** EMG values with eyes closed versus eyes open for the two groups.

sEMG sum	EC			EO				
	*N*	Mean	SD	*N*	Mean	SD	*t* value	*p* value
TMD	20	18.88	0.5875	20	20.55	0.6875	8.258565	≤0.001
Healthy	20	12.84	0.4375	20	12.95	0.4625	0.772703	0.444

**Table 4:** EMG values with eyes closed and with glasses on.

Muscle group		CE			CEWG				
		*N*	Mean	SD	*N*	Mean	SD	*t* value	*p* value
TMD group	LTA	20	2.65	0.5	20	2.55	0.6	0.573	0.570
	RTA	20	2.78	0.8	20	2.69	0.6	0.402	0.690
	LMM	20	2.02	0.6	20	2.12	0.5	0.573	0.570
	RMM	20	2.21	0.5	20	2.31	1.1	0.370	0.713
	LSM	20	2.36	0.8	20	2.45	0.6	0.402	0.690
	RSM	20	2.87	0.6	20	2.77	1.1	0.357	0.723
	LDA	20	2.01	0.5	20	2.1	1.1	0.333	0.741
	RDA	20	1.98	0.4	20	1.48	0.8	2.500	0.017

Healthy group	LTA	20	1.54	0.5	20	1.54	0.6	0.000	1.000
	RTA	20	1.63	0.4	20	1.69	0.9	0.272	0.787
	LMM	20	1.78	0.4	20	1.71	1.1	0.267	0.791
	RMM	20	1.81	0.6	20	1.56	0.5	1.431	0.160
	LSM	20	1.95	0.8	20	1.89	1	0.210	0.835
	RSM	20	1.85	0.3	20	1.85	0.8	0.000	1.000
	LDA	20	1.13	0.3	20	1.12	0.6	0.067	0.947
	RDA	20	1.15	0.2	20	1.17	0.5	0.166	0.869

**Table 5:** EMG values with eyes closed with and without glasses on.

sEMG sum	EC			ECWG				
	*N*	Mean	SD	*N*	Mean	SD	*t* value	*p* value
TMD group	20	18.88	0.5875	20	18.47	0.8	1.847338	0.072
Healthy group	20	12.84	0.4375	20	12.53	0.75	1.59668	0.119

**Table 6:** EMG values of the individual muscles with eyes open with and without glasses on.

Muscle group		EO			EOWG				
		*N*	Mean	SD	*N*	Mean	SD	*t*.value	*p* value
TMD group	LTA	20	2.83	0.7	20	3.95	0.9	4.393	≤0.001
	RTA	20	2.94	0.8	20	4.78	1	6.426	≤0.001
	LMM	20	2.37	0.8	20	3.12	0.9	2.785	0.008
	RMM	20	2.53	0.6	20	2.41	0.8	0.537	0.595
	LSM	20	2.62	0.7	20	3.66	1.8	2.408	0.021
	RSM	20	2.95	0.7	20	3.57	1.1	2.127	0.040
	LDA	20	2.26	0.6	20	3.11	0.5	4.867	≤0.001
	RDA	20	2.05	0.6	20	3.38	0.9	5.499	≤0.001

Healthy group	LTA	20	1.55	0.5	20	1.71	0.2	1.329	0.192
	RTA	20	1.64	0.3	20	1.58	0.8	0.314	0.755
	LMM	20	1.79	0.4	20	1.58	0.3	1.878	0.068
	RMM	20	1.82	0.5	20	1.51	0.6	1.775	0.084
	LSM	20	1.97	0.9	20	1.85	0.2	0.582	0.564
	RSM	20	1.86	0.5	20	1.92	0.5	0.379	0.706
	LDA	20	1.16	0.4	20	1.16	0.6	0.000	1.000
	RDA	20	1.16	0.2	20	1.17	0.3	0.124	0.902

**Table 7:** EMG values with open eyes with and without glasses.

sEMG values	EO			EOWG				
	*N*	Mean	SD	*N*	Mean	SD	*t*-value	*p*-value
TMD group	20	20.55	0.6875	20	27.98	0.9875	27.61517	≤0.001
Healthy group	20	12.95	0.4625	20	12.48	0.4375	3.30155	0.002

## Data Availability

The data used to support the findings of this study have not been made available because they are private.
